# A rare case of primary invasive urothelial carcinoma of the renal pelvis and calyx: a case report

**DOI:** 10.3332/ecancer.2025.1928

**Published:** 2025-06-20

**Authors:** Olatunde Olaniyi Abiodun Oluwafemi, Eberechukwu Uchime Kasiemobi, Mustapha Babatunde, Bankole Kofi Adedeji, Oyelayo Oluwaseun Olaoluwa, Ismaheel Aderogba Azeez, Ezekpo Okechukwu Obumneme

**Affiliations:** 1 Department of Anatomic Pathology and Forensic Medicine, Afe Babalola University, PMB 5454, Ado-Ekiti, Ekiti State, 3601102, Nigeria; 2 Histopathology Department, Federal University of Health Sciences, PMB 145, Otukpo, Benue State, 972101, Nigeria; a https://orcid.org/0000-0002-4044-3418

**Keywords:** upper urinary tract, urothelial carcinoma, misdiagnosis

## Abstract

Upper urinary tract urothelial carcinomas (UTUCs) are rare malignant neoplasms, representing about 5% of all urothelial carcinomas (UCs). The incidence of primary UTUC in the renal pelvis and calyx is quite rare. UTUC is a high-grade tumour with a poor prognosis at presentation. Characteristically, UTUC presents with symptoms such as gross and microscopic hematuria or flank pain. Its mode of definitive diagnosis remains histopathology examination, despite using computed tomography urography (CTU) as the gold imaging standard. However, atypical clinical presentations and abnormal radiologic findings could lead to misdiagnosis of UTUC.

We report a 59-year-old male who presented with recurrent left flank pain of 2 years duration, and an episode of hematuria. A CTU showed no classic radiologic feature of upper UCs; however, his abdominal magnetic resonance imaging was suggestive. He subsequently had a left radical nephroureterectomy. The post-operative histology report showed a primary invasive high-grade UC of the left renal pelvis and calyx. He was counseled on the findings and placed on surveillance. There are few reported cases of UTUC of the renal pelvis and renal calyx; given that it is a rare malignancy. This is quite concerning, especially with the missed imaging finding by CTU.

## Introduction

Urothelial carcinoma (UC) is the most common primary neoplasm arising from the epithelial lining of the urinary tract system. It is the most common tumour from the urinary bladder, accounting for 90% to 95% of the urinary tract UC [1]. However, UC arising from the upper urinary tract is rare in occurrence, accounting for about 5% to 10% and even the rarer form found in the ureter, with less than 1% [1–3].

According to GLOBOCAN 2022 data, bladder carcinoma is the ninth most common cancer worldwide, with 614,000 new cases and 220,000 deaths. Urothelial bladder cancer (UCB) has the highest incidence in developed nations such as the United States of America and Europe, compared to the Middle East, Asia and Africa, most especially in Sub-Saharan Africa. The incidence of the upper urinary tract urothelial carcinoma (UTUC) is one to two per 100,000 persons, with a higher incidence in the developed nations compared to the developing nations. Both UTUC and UCB are histologically similar and are often termed UC. Clinically, UTUC presents as gross hematuria in 70%–80% of cases, other symptoms include flank pain and flank mass or renal colic. Globally, the incidence of UTUC arising from the renal pelvis is two to three times more common in men compared to women [1, 2, 4–7].

Although both UCB and UTUC share similar histopathologic features with several risk factors (genetic and environmental, especially use of tobacco), they significantly differ in terms of molecular and clinical characteristics. The recent genomic study has reported an association between Lynch syndrome and UTUC. There is a higher recurrence rate of 22%–47% following recommended surgical treatment of UTUC in the bladder, as compared to a lower value of developing UTUC following primary UCB surgical treatment [7, 8]. In a cohort of 1, 529 patients who were treated for primary superficial bladder cancer, the incidence of UTUC was only 2.6% [8]. A study has also reported a 2%–6% recurrence rate of UTUC patients developing contralateral upper urinary malignancy due to UC [9]. The overall prognosis of UTUC is poor, as about 60% of the cases at the time of diagnosis had undergone metastasis compared to 20% to 25% of patients diagnosed with UCB [10, 11]. The monoclonal theory concerning the origin of UTUC is that the intraepithelial seedling along the entire urinary system is well accepted as against the carcinogenic hit theory [2, 7, 12].

In Sub-Saharan Africa, there is a paucity of data on the incidence of UTUC of the renal pelvis and renal calyx. This is probably a result of the low index of suspicion, lack of data across cancer registries, lack of clinical expertise and high cost of diagnostic imaging techniques [2]. There is no previously reported case of primary invasive high-grade UC of the renal pelvis and renal calyces without a previous diagnosis of UC of the bladder in Nigeria. This will be the first case and we are reporting the case to increase awareness among clinicians for a high index of suspicion and to improve patients’ care. We present a rare case of a 59-year-old male with primary invasive high-grade UC of the renal pelvis and renal calyx.

## Case report

A 59-year-old male presented with complaints of recurrent left flank pain for a 2-year duration. The pain was said to be insidious in onset and intermittent. There was also an episode of hematuria, which was total but had resolved spontaneously; however, there were no other urinary symptoms. He is a known hypertensive and consumes alcohol.

**Figure 1.**
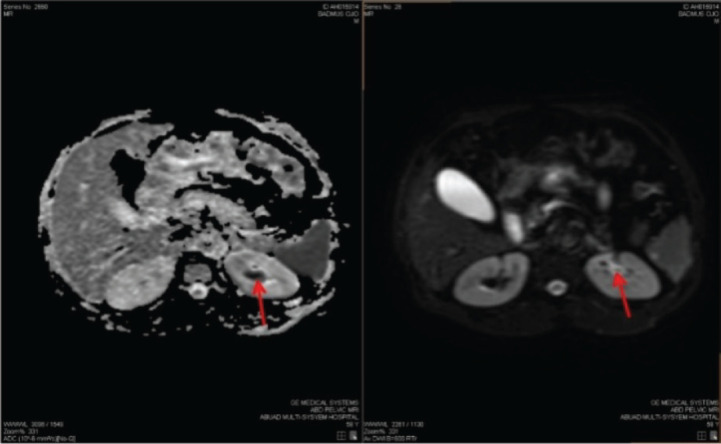
MRI. DWI and ADC images showing a focal area of diffusion restriction in the left upper pole calyx (red arrow).

**Figure 2.**
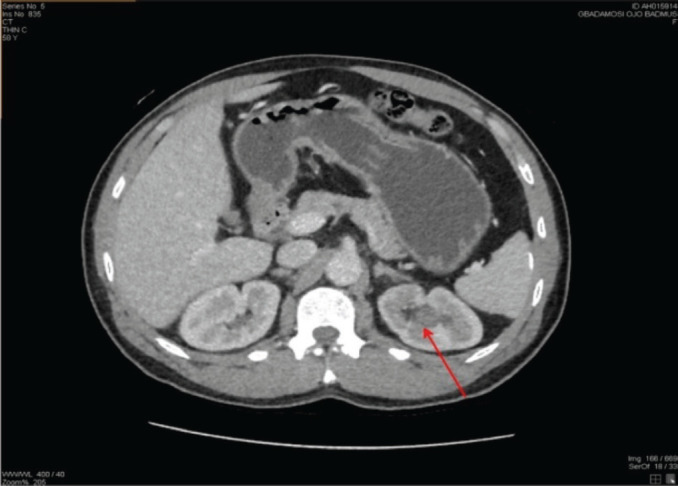
A repeated contrast-enhanced CTU image (axial section) showing a focal, mildly enhancing tumour seen in the left upper pole calyx (red arrow).

His physical examination showed a middle-aged man, in painful distress, afebrile, not pale and well hydrated. His abdominal examination revealed tenderness on the left flank, with no detected palpable mass. An Abdominopelvic ultrasound scan and computed tomography urography (CTU) initially detected no abnormality; while an abdominopelvic magnetic resonance imaging (MRI) (Figure 1) report was suggestive of a growth in the upper pole involving the collecting system of the left kidney. Following receipt of the abdominopelvic MRI report, a review of the CTU was done, which showed a focal, mildly enhancing tumour at the left upper pole calyx (Figure 2). The right kidney appeared normal on both CTU and abdominopelvic MRI (Figures 1 and 2).

**Figure 3.**
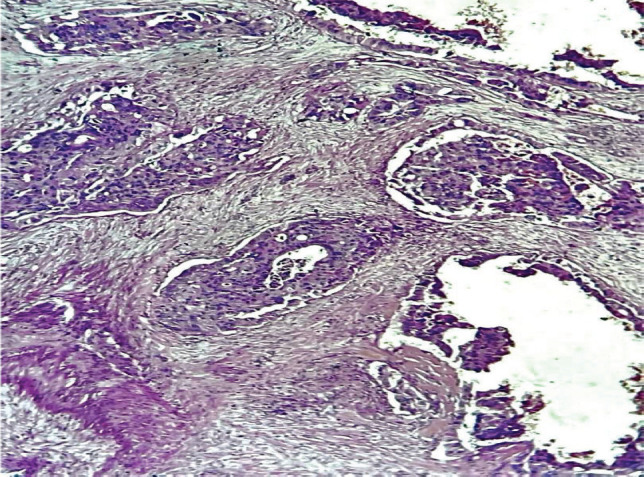
Photomicrograph showing malignant urothelial cells disposed in nesting pattern within the renal pelvis and calyces, invading adjacent renal parenchyma, with areas of vascular invasion. (×400 magnification).

He had a left radical nephroureterectomy (RNU) done with intra-operative findings of a grossly normal left kidney. The histology reported an invasive high-grade urothelial tumour of the renal pelvis and calyces with lympho-vascular invasion (Figure 3). He was then counseled and placed on surveillance. However, 9 months post-surgery, he presented with recurrent left flank pain and a CT scan of his abdomen revealed growth at the left renal bed, suggestive of recurrence, with multiple liver metastases. The patient is currently on palliative chemotherapy.

## Discussion

Upper urinary tract carcinoma is a rare tumour of the genitourinary system. The diagnosis of UTUC is often done through radiology imaging techniques, either following symptomatic or asymptomatic presentations [10]. Worldwide, studies have shown that CTU is the most reliable and dependable imaging technique with a sensitivity and specificity of 67%–100% and 93%–99%, respectively [13, 14]. However, there are reports of CTU failure to identify UTUC presenting with atypical symptoms and signs, resulting in missed diagnosis or misdiagnosis [15].

Globally, the gold standard of treatment for UTUC is RNU with bladder cuff excision using either robotic, open or laparoscopic technique [5]. However, UTUC patients treated with RNU has a poor survival rate, with bladder tumour recurrence rates of 22%–47%, thus necessitating the clinician’s use of neoadjuvant, adjuvant or immunotherapy to improve outcome [10, 16, 17]. 

In this study, the patient presented with symptoms of gross hematuria and left flank pain and on admission, his urinalysis showed gross hematuria. Despite these clinical findings, the detection of the left renal tumour was initially missed using CTU. The tumour was later detected on CTU after a follow-up abdomino-pelvic MRI scan had suggested a lesion in the upper pole of the left kidney. The diagnosis was confirmed post operatively with the pathology report of an invasive high-grade UC of the left renal pelvis and renal calyx. Clinicians should, therefore, have a high index of suspicion in diagnosing upper urinary tract carcinoma, especially in patients presenting with typical features and advanced imaging techniques such as MRI could be complimentary to the CTU gold standard. The utilisation of urine cytology, retrograde ureteropyelogram as well as ureteroscopy and renoscopy in the assessment of such patients would have assisted in locating the lesion in the renal pelvis and biopsy taken for pathological examination. Multi-disciplinary team meetings are useful for discussing the diagnosis and management of such patients, with patient involvement in decisions related to the management options [2].

Studies have reported that a substantial number of patients who had RNU due to a non-functional kidney as a result of urolithiasis were subsequently diagnosed with a malignancy [18]. The index case has no clinical symptoms suggestive of an infective cause. The laboratory work-up reported a normal complete blood count and his renal function tests were essentially normal. However, this does not rule out the possibility of misdiagnosing an upper urinary tract carcinoma based on the intra-epithelial seedling hypothesis.

Studies have documented the peak age incidence of 70–90 years for UTUC. However, the index case presented at 59 years of age, which could be responsible for the missed diagnosis due to the low index of suspicion [19].

Recent genomic studies have associated Lynch syndrome also known as hereditary non-polyposis colorectal carcinoma, with 21% of UTUC diagnosed cases. Lynch syndrome is an autosomal dominant disorder associated with a DNA mismatch repair gene mutation. Several tumours have been associated with UTUC, which is ranked as the third commonest. The index patient could not undergo genomic studies, thus, the probability of a genetic cause could not be ascertained [20, 21]. 

A known paraneoplastic syndrome called tumour-associated leucocytosis has been reported in UC with a prevalence of about 1%. This condition has been associated with advanced malignancy, with the consequence of poor prognosis. This index case presented with a normal white blood cell count, thus probably suggesting a non-paraneoplastic origin [22, 23].

The definitive diagnosis of UTUC remains the histological examination of the tumour in assessing the tumour stage and grade. Clinicians must be on high alert to prevent a missed diagnosis of UTUC of the renal pelvis involving the upper pole of the kidney, most especially when CTU imaging standard fails and clinical presentation are typical [2, 24]. 

## Conclusion

Upper urinary tract carcinoma of the renal pelvis and renal calyx is a rare malignancy, with an aggressive tendency and poor prognosis at diagnosis. There are very few reports in the literature till date on UTUC of the pelvis in Sub-Saharan Africa [2]; therefore, future research should prioritise standardising imaging diagnostic modalities and understanding tumour biology. This report has contributed to the knowledge of UTUC and aided diagnostic accuracy of the disease.

## Conflicts of interest

The authors have no conflicts of interest to declare.

## Funding

This research received no specific grant from funding agencies in the public, commercial or not-for-profit sectors.

## Informed consent

Informed consent was obtained from the patient to use the patient’s data and clinical information in a journal article. The written informed consent can be confidentially delivered to the journal editor on request.

## Ethical approval

The Health Research and Ethics Committee of Afe Babalola University Ado-Ekiti Multisystem Hospital, Ekiti State, Nigeria, approved this study with reference number AMSH/REC/25/005. See Appendix A. The article also maintained confidentiality.

## Author contributions

OOAO, UKE, MB, BKA, OOO, IAA and EOO participated in data acquisition through clinical management, pathlogical and radiological analysis and data analysis and interpretation. OOAO drafted the manuscript while UKE, MB, BKA, OOO, IAA and EOO revised the manuscript for sound intellectual content. All the authors approved the final version of the manuscript.
